# First detection of *Ehrlichia minasensis* in *Hyalomma marginatum* ticks collected from cattle in Corsica, France

**DOI:** 10.1002/vms3.140

**Published:** 2019-01-21

**Authors:** Vincent Cicculli, Shirley Masse, Lisandru Capai, Xavier de Lamballerie, Remi Charrel, Alessandra Falchi

**Affiliations:** ^1^ EA7310 BIOSCOPE Laboratoire de Virologie Université de Corse‐Inserm Corte France; ^2^ Unité des Virus Emergents (UVE) Aix‐Marseille Université IRD 190 INSERM 1207 IHU Méditerranée Infection Marseille France

**Keywords:** *Ehrlichia minasensis*, *Hyalomma marginatum*, Corsica, France

## Abstract

Ehrlichiosis are severe, feverish tick‐borne illnesses caused by specific species within the genus *Ehrlichia* (Anaplasmataceae family). Recent data suggest that ruminants in Corsica area reservoir for several Anaplasmataceae species. The purpose of our study was to determine whether *Ehrlichia* species could be detected in ticks collected in Corsican ruminants by using molecular methods. Ticks were collected in northern Corsica: (i) in May 2016 from sheep bred in one farm located in a 5000‐inhabitants village and (ii) from cattle in June and July 2016 in a slaughterhouse. There sheep and cattle whole skin was inspected and ticks were collected manually. A total of 647 ticks was collected in northern Corsica during this study: 556 (86%) belonged to the *Rhipicephalus bursa* species and 91 (14%) to *Hyalomma marginatum*. The 91 *H*. *marginatum* ticks were organized in 27 pools, of which one (3.7%) was found positive for the presence of *E*. *minasensis*; this pool consisted of six ticks collected from a cow bred and raised northwestern Corsica. Ehrlichial DNA was not detected in *R*. *bursa* ticks. The 16S rRNA and *groEL* gene sequences of *Ehrlichia* detected in the *H*. *marginatum* pool showed 100% (303/303 bp) and 99.8% (555/556) of nucleotide identity with *E*. *minasensis,* respectively. Phylogenetic analyses demonstrated the highest closeness with *E*. *minasensis* UFMG‐EV genotype than to any other *E*. *canis* strains. To our knowledge, this is the first report of *E*. *minasensis* outside of Brazil, Ethiopia and Canada. This identification of *E*. *minasensis* in *H*. *marginatum* merits to be further investigated and pleads for translational studies addressing the potential impact of vector‐borne diseases of human and veterinary impact through large‐scale research and surveillance programmes in Corsica.

## Introduction

Ehrlichiosis are severe, feverish tick‐borne illnesses caused by specific species within the genus *Ehrlichia* (Anaplasmataceae family) (Nicholson *et* *al*. 2010). The genus *Ehrlichia* consists of *E*. *chaffeensis, E*. *canis, E*. *ewingii, E*. *muris* and *E*. *ruminantium,* all of which are capable of causing infections in both humans and domestic animals (Rar & Golovljova 2011; Vieira *et* *al*. 2011). *Ehrlichia minasensis* is a recently described species most closely related with although clearly distinct from *E*. *canis*. *Ehrlichia minasensis* (i) was discovered in naturally infected dairy cattle and mule deer in Canada (genotype BOV2010) (Gajadhar *et* *al*. 2010), (ii) was detected in the haemolymph of Brazilian *Rhipicephalus microplus* ticks (*Ehrlichia* sp. UFMG‐EV) (Cabezas‐Cruz *et* *al*. 2016), (iii) isolated in Brazil (strain UFMT‐BV), where it proved to be pathogenic for cattle (Aguiar *et* *al*. 2014) and (iv) isolated (strain UFMG‐EV) from a partially engorged *R*. *microplus* female tick (Cabezas‐Cruz *et* *al*. 2016). *Ehrlichia minasensis* was established as a new species in 2016. *Ehrlichia minasensis* can be grown in *Ixodes scapularis* cell lines (ID8) and dog macrophages (DH82) (Cabezas‐Cruz *et* *al*. 2016). Corsica is a French Mediterranean island characterized by a warm‐summer Mediterranean climate with a high variability of microclimates because of peculiar geographical situation (Grech‐Angelini *et* *al*. 2016). Corsican livestock farming (sheep, goats, pigs, and cattle) is mainly of the extensive type; thus frequent interactions between livestock, wildlife and human populations favour the circulation of ticks and tick‐borne microorganisms (Grech‐Angelini *et* *al*. 2016).

Recent data suggest that ruminants in Corsica area reservoir for several Anaplasmataceae species (Dahmani *et* *al*. 2017b). *Ehrlichia canis* was detected once in Corsica in a non‐engorged *R*. *bursa* tick collected from a cow (Dahmani *et* *al*. 2017b). The purpose of our study was to determine whether other *Ehrlichia* species could be detected in ticks collected in Corsican ruminants by using molecular methods.

## Materiel and methods

Ticks were collected in northern Corsica: (i) in May 2016 from sheep bred in one farm located in Corte, a 5000‐inhabitants village and (ii) from cattle in June and July 2016 in the Ponte‐Leccia slaughterhouse. There sheep and cattle whole skin was inspected and ticks were collected manually. They were identified at the species level based on taxonomic keys and morphometric tables using a binocular microscope (Estrada‐Pena *et* *al*. 2004). Morphologic identification was confirmed by mitochondrial 16S rDNA sequence analysis (Table 1) (Black & Piesman 1994). Ticks were washed once in 70% ethanol for 5 min and twice in distilled water for 5 min. Ticks collected from sheep were analysed individually. Pools consisting of 2–6 ticks collected from cattle (same species, same animal) were analysed. Ticks were crushed using the TissueLyser II (Qiagen, Hilden, Germany) in a phosphate‐buffered saline solution at 2800 *g* for 20 s. DNA extraction was performed on a QIAcube HT (Qiagen) using QIAamp Cador Pathogen Mini kit according to the manufacturer's instructions. DNA was eluted in 150 *μ*l of buffer and stored at −20°C. Samples were analysed by using a qPCR for detection of *Ehrlichia* spp. specific including a positive (*Ehrlichia_*spp.) and a negative control (distilled water) (genesig^®^ Standard Kit). *Ehrlichia* DNA was also identified by conventional PCR using (i) tick‐borne Anaplasmataceae specific primers targeting a 345‐bp region of the 16S rRNA (Parola *et* *al*. 2001) and (ii) genus‐specific primers targeting a 590‐bp region of the heat shock protein (*groEL*) gene (Dahmani *et* *al*. 2017a). PCR conditions and primer sequences are described in Table 1. The reactions were carried out using a GeneAmp PCR Systems 9700 Applied Biosystems (Courtaboeuf, France). The PCR products were UV light‐visualized in 2% agarose gel in Tris‐Acetate‐EDTA (TAE Buffer) after staining with ethidium bromide. Sequences obtained in this study were deposited in the GenBank using the National Center for Biotechnology Information (NCBI) BankIt 3.0 submission tool (https://www.ncbi.nlm.nih.gov/WebSub/) (accession number for *H*. *marginatum* MH663977‐83 and for *R*. *bursa* MH663984‐90). Sequences of *Ehrlichia* 16S rRNA and *groEL* genes correspond to acc. nos MH657222 and MH675614 respectively. All sequences were assembled and compared with selected homologous sequenced retrieved from the GenBank nucleotide database using BLASTn (Altschul *et* *al*. 1997). Each model was inferred using the Maximum Likelihood method implemented in Mega X (Kumar *et* *al*. 2018). The bootstrap consensus tree was conducted with 1000 replicates (Felsenstein 1985).

**Table 1:** Primers and probes used in this study

Species	Target	Name	Sequence	Cycles	References
Conventional PCR*
*Ehrlichia* ssp.	*groEL*	*Ehr‐ groel‐ F*	GTTGAAAARACTGATGGTATGCA	95°C 5 min, 40 × [95°C 60 s, 50°C 60 s, 72°C 60 s], 72°C 7 min	(Dahmani *et* *al*. 2017a)
		*Ehr‐ groel‐ R*	ACACGRTCTTTACGYTCYTTAAC		
	*16S rRNA* **	*Ehr‐16S‐D*	GGTACCYACAGAAGAAGTCC	95°C 5 min, 40 × [95°C 60 s, 55°C 60 s, 72°C 60 s], 72°C 7 min	(Parola *et* *al*. 2001)
		*Ehr‐16S‐R*	TAGCACTCATCGTTTACAGC		
Ticks	16S rDNA	*16S+1*	CTGCTCAATGATTTTTTAAATTGCTGTGG	95°C 5 min, 10 × [92°C 60 s, 48°C 60 s, 72°C 90 s], 32 × [92°C 60 s, 54°C 35 s, 72°C 90 s],72°C 7 min	(Black & Piesman 1994)
		*16S‐1*	CCGGTCTGAACTCAGATCAAGT		

* For each PCR reaction, the template DNA had a final concentration <200 ng.

**Primers designed to amplify a fragment of the 16S rRNA gene from bacteria within the family of Anaplasmatace.

The pathogens detected in pools were expressed as the percentage and minimum infection rate based on the assumption that each PCR‐positive pool contained at least one positive tick (Sosa‐Gutierrez *et* *al*. 2016).

## Results and discussion

A total of 647 ticks was collected during this study; 586 ticks were taken from 42 cows, and the remaining 61 ticks were removed from 60 sheep. In cattle, *R*. *bursa* was the most abundant species (n = 495; 84.5%), followed by *Hyalomma marginatum* (n = 91; 15.5%). Ticks collected from sheep all belonged to the *R*. *bursa* species. The 586 ticks collected in cattle were organized as 127 pools consisting of 100 pools of *R*. *bursa* and 27 pools of *H*. *marginatum*. Ehrlichial DNA was detected neither in the 61 *R*. *bursa* ticks from sheep, not in the 100 pools of *R*. *bursa* collected from cattle.

**Figure 1:**
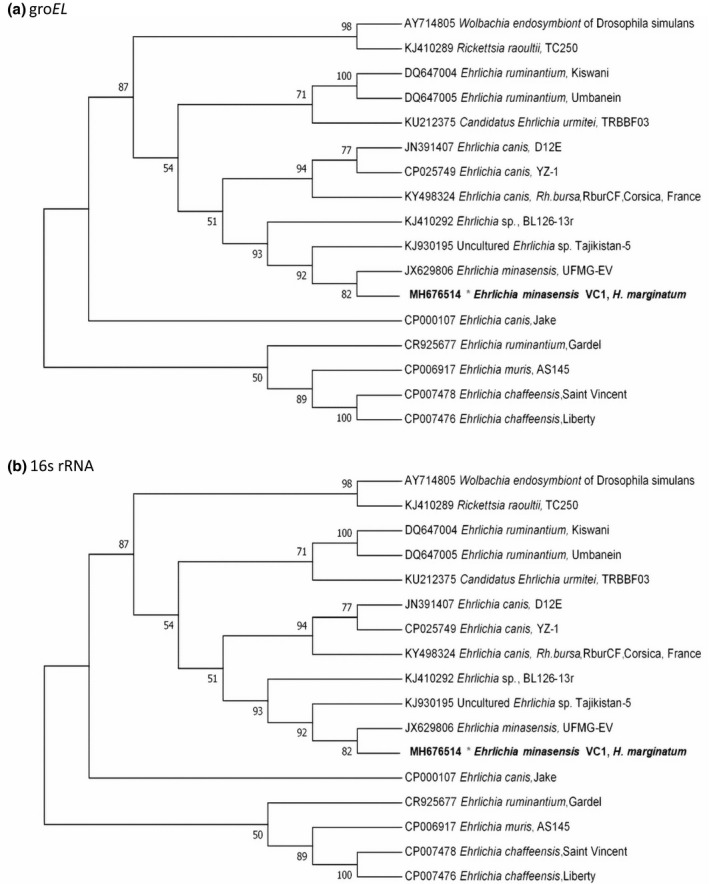
Phylogenetic position of *Ehrlichia minasensis* detected in the *Hyalomma marginatum* pool collected on cattle in Corsica using *groEL* (a) and 16S rRNA (b). All sequences were assembled and compared with homologous sequenced retrieved from the GenBank nucleotide database using BLASTn (Altschul *et* *al*. 1997). The Hasegawa–Kishino–Yano and the Tamura 3‐parameter models were identified as the best‐fit models under the Akaike Information Criterion, for 16Sr RNA and *groEL* sequences respectively. Each model was inferred using the Maximum Likelihood method implemented in Mega X (Kumar *et* *al*. 2018). The bootstrap consensus tree was conducted with 1000 replicates (Felsenstein 1985).

Ehrlichial DNA (16S rRNA and *groEL*) was detected in one of the 27 pools (3.7%) of *H*. *marginatum* ticks collected from cattle. This pool consisted of six ticks collected from an animal raised in a county of northwestern Corsica. The 16S rRNA sequence of *Ehrlichia* showed 100% identity (303/303 bp) with *E*. *minasensis* strain (JX629805) and 99% of identity with *E*. *canis* TWN (GU810149.1). The 556‐bp *groEL* gene sequence showed 99.8% identity (555/556 bp) with the homologous sequence of *E*. *minasensis* strain UFMG‐EV (JX629806) and 97% of identity (521/554 bp) with the *E*. *canis* strain previously described in Corsica (KY498324). These values were closed to those obtained by comparing the 16S rRNA and *groEL* gene sequences of *Ehrlichia* sp. UMFG‐EV to *E*. *canis* TWN (98.3% and 97.2% respectively) (Cabezas‐Cruz *et* *al*. 2016). Phylogenetic analyses proved that our Corsican strain was most closely related with *E*. *minasensis* UFMG‐EV than to *E*. *canis* strains (Fig. 1) (Cabezas‐Cruz *et* *al*. 2016).

To the best of our knowledge, this is the first description of *E*. *minasensis* in Corsica after recent identification in the Americas (Cabezas‐Cruz *et* *al*. 2016). In agreement with previous phylogenetic analyses, we observed that *E*. *minasensis* is a sister taxa of *E*. *canis*. This is also the first description of *E*. *minasensis* in *H*. *marginatum* tick. So far, it has been reported in haemolymph of *R*. *microplus* ticks (Cabezas‐Cruz *et* *al*. 2016)*,* and in blood of an apparently healthy cattle in Ethiopia, where *R*. *microplus* is not described (Hailemariam *et* *al*. 2017). Interestingly, *H*. *marginatum* is present in Ethiopia (ECDC.EUROPA.EU, 2018).

The role of *H*. *marginatum* in the transmission of *E*. *minasensis* remains unknown. The presence of a bacterium in an engorged tick could be due to the presence in the blood meal. The presence of *H*. *marginatum* in Corsica, endemic in southern Europe, was previously reported (Matsumoto *et* *al*. 2004; Grech‐Angelini *et* *al*. 2016). At the outset of this study, *H*. *marginatum* ticks collected in Corsica from ruminants had never been detected positive through PCR for microorganisms belonging to Anaplasmataceae (Dahmani *et* *al*. 2017b). Identification of *E*. *minasensis* in *H*. *marginatum* merits to be further investigated and pleads for translational studies addressing the potential impact of vector‐borne diseases of human and veterinary impact through large‐scale research and surveillance programmes in Corsica.

## Source of funding

This work was supported by the Corsican Territorial Collectivity and the University of Corsica.

## Conflicts of interest

The authors of the work have no conflict of interests to disclose.

## Ethical statement

No ethical approval was required, as this study does not involve clinical trials or experimental procedures. The cattle inspected were slaughtered for human consumption. The slaughterhouse staff gave permission to collect ticks from the whole skins of animals. This study did not involve endangered or protected species.

## Contributions

VC and AF conceived the study,analysed data and draft the manuscript . SM microbiological diagnosis of bacteria. CL and VC collected ticks. XdL and RC draft the manuscript.
